# Validation of the online PREDICT tool in a cohort of early breast cancer in Brazil

**DOI:** 10.1590/1414-431X2022e12109

**Published:** 2022-11-04

**Authors:** M.B. Magário, R.R. dos Santos, L.A. Teixeira, D.G. Tiezzi, F.F. Pimentel, H.H.A. Carrara, J.M. de Andrade, F.J. Candido dos Reis

**Affiliations:** 1 Departamento de Ginecologia e Obstetrícia, Faculdade de Medicina de Ribeirão Preto, Universidade de São Paulo, Ribeirão Preto, SP, Brasil

**Keywords:** Breast cancer, Prognosis model, Survival, PREDICT

## Abstract

PREDICT is a tool designed to estimate the benefits of adjuvant therapy and the overall survival of women with early breast cancer. The model uses clinical, histological, and immunohistochemical variables. This study aimed to evaluate the model's performance in a Brazilian population. We assessed the discrimination and calibration of the PREDICT model to estimate overall survival (OS) in five and ten years of follow-up in a cohort of 873 women with early breast cancer diagnosed from January 2001 to December 2016. A total of 743 patients had estrogen receptor (ER)-positive and 130 had ER-negative tumors. The area under the receiver operating characteristic (ROC) curve (AUC) for discrimination was 0.72 (95%CI: 0.66–0.78) at five years and 0.67 (95%CI: 0.61–0.72) at ten years for women with ER-positive tumors. The AUC was 0.72 (95%CI: 0.62–0.81) at five years and 0.67 (95%CI: 0.54–0.77) at ten years for women with ER-negative tumors. The predicted survival in ER-positive tumors was 91.0% (95%CI: 90.2–91.6%) at five years and 79.3% (95%CI: 77.7–81.0%) at ten years, and the observed survival 90.7% (95%CI: 88.6–92.9%) and 77.2% (95%CI: 73.4–81.4%), respectively. The predicted survival in ER-negative tumors was 84.5% (95%CI: 82.5–86.6%) at five years and 75.0% (95%CI: 71.6–78.5%) at ten years, and the observed survival 76.3% (95%CI: 69.1–84.3%) and 67.9% (95%CI: 58.6–78.6%), respectively. In conclusion, PREDICT was accurate to estimate OS in women with ER-positive tumors and overestimated the OS in women with ER-negative tumors.

## Introduction

Breast cancer is the malignant neoplasm with the highest incidence in women in Brazil. The estimated incidence for the triennium 2020-2023 is 61 per 100,000 women or a total of 66,280 new cases annually (1). Over the past three decades, the combination of screening and adjuvant treatment has led to a dramatic increase in survival for women with breast cancer (2,3).

Breast cancer is a heterogeneous disease. Multiple approaches have been developed to characterize the disease in subgroups for specific treatments. Clinical presentation, histopathological tumor features, biomarkers, and multigene panels are valuable tools for predicting treatment effects and prognosis and improving personalized treatment (4). However, the algorithms for clinical decisions are complex, and some tests are not available in middle- and low-income countries.

PREDICT is an online tool developed in the United Kingdom for estimating the prognosis and benefit of adjuvant treatment after surgery for early breast cancer (5). The model is available online (6), free for health professionals and patients, translated into several languages, and validated in multiple populations (7). PREDICT uses clinical, pathological, and immunohistochemistry variables widely implemented in clinical practice worldwide.

Our study aimed to validate the PREDICT tool in a cohort of patients with early breast cancer in Brazil. We assessed the discrimination and the calibration of the model to estimate the overall survival five and ten years after the surgery.

## Material and Methods

### Study design

We evaluated a historical cohort of patients with early breast cancer treated with primary surgery at Hospital das Clínicas of Ribeirão Preto Medical School.

### Patient selection

We assessed all medical records of breast cancer patients treated in our hospital from 2001 to 2016 (to guarantee at least 5 years of follow-up). Then, we selected the patients treated with primary surgery. We excluded patients without documented follow-up or adjuvant treatment. The informed consent was waived.

### Variables

The following variables were collected: age at the surgery, menopausal status, detection mode (screening *vs* symptoms), tumor size (mm), tumor grade, number of positive axillary lymph nodes, estrogen receptor status, HER-2 marker status, Ki-67 marker status, type of treatment received, current condition (living or dead), survival time in days, and cause of death.

### Statistical analysis

We used the software R (version 4.0.2 (2020-06-22)) for all analyses. We used the Kaplan-Meyer estimator and Cox regression for survival analysis. The survival time was censored at 10 years of follow-up, and the event was death from any cause. The prognostic index (PI) for each patient was calculated as the sum of the weighted prognostic factors as proposed in version 2.1 of the PREDICT model (7), the non-breast cancer mortality index (MI) was a function of the patient's age at diagnosis [0.0698252 × ((age/10)^2^ - 34.23391957)], and the treatment effect (TE) was calculated as the sum of weighted treatment factors.

The breast cancer specific survival (bS) was calculated as exp (-exp (baseline breast cancer survival at time t + PI + TE)) and other causes specific survival (oS) as exp (-exp (baseline other cause specific survival + MI)). The overall estimated survival assuming independent competing risk and treatment combination at time t (S) was calculated by multiplying bS by oS.

We assessed the models' discrimination with the Royston D (8), dichotomized Kaplan Meyer curves, and receiver operating characteristic (ROC) curves. Royston D measures the prognostic separation of survival curves and can be interpreted as the log hazard ratio that compares two equal-sized risk groups defined by dichotomizing the distribution of the patient prognostic indices at the median value (9).

We assessed the model calibration by tabulating the predicted and observed survival at five and ten years of follow-up according to ER tumor status, age at diagnosis, tumor size, positive lymph nodes, and adjuvant therapy. We also calculated the Integrated Calibration Index (ICI), equivalent to the mean difference between predicted probabilities and observed probabilities derived from a smoothed calibration curve (10). We plotted the curves for the COX proportional hazard model using restricted cubic splines to calculate the calibration curves and the density function to estimate the distribution of predicted risk across the samples (11).

## Results

### Characteristics of the patients

A total of 873 women with breast cancer who underwent primary surgery from January 2001 to December 2016 were included in the study. Table 1 shows the baseline characteristics of the patients. A total of 743 cases had tumors positive for estrogen receptor (ER) and 130 had ER-negative tumors.

**Table 1:** Baseline characteristics of the cohort.

Variable	ER-Negative	ER-Positive	P
Number of cases	n=130	n=743	
Age in years (mean, SD)	55.6 (13.1)	58.5 (12.7)	0.019
Menopause			0.368
No	46 (35.4%)	230 (31.0%)	
Yes	84 (64.6%)	513 (69.0%)	
Detected by			0.071
Screening	35 (26.9%)	276 (37.1%)	
Symptoms	94 (72.3%)	459 (61.8%)	
Unknown	1 (0.8%)	8 (1.1%)	
Tumor size in mm (mean, SD)	26.4 (14.0)	21.1 (12.8)	<0.001
Breast surgery			0.597
Conservative surgery	84 (64.6%)	501 (67.4%)	
Mastectomy	46 (35.4%)	242 (32.6%)	
Axillary surgery			0.141
Axillary dissection	72 (55.4%)	344 (46.3%)	
None	0	2 (0.3%)	
Sentinel biopsy	58 (44.6%)	397 (53.4%)	
Tumor histology			0.013
Ductal invasive	121 (93.1%)	643 (86.5%)	
Lobular invasive	1 (0.8%)	42 (5.7%)	
Mixed invasive	0	2 (0.3%)	
Non-classified carcinoma	1 (0.8%)	0	
Special type carcinoma	7 (5.4%)	56 (7.5%)	
Tumor grade			<0.001
1	3 (2.3%)	240 (32.3%)	
2	61 (46.9%)	403 (54.2%)	
3	66 (50.8%)	100 (13.5%)	
Positive lymph nodes (median, interquartile range)	0.0 [2.0]	0.0 [1.0]	0.073
Her2 status			<0.001
Negative	86 (66.2%)	630 (84.8%)	
Positive	43 (33.1%)	112 (15.1%)	
Unknown	1 (0.8%)	1 (0.1%)	
Ki67 status			0.634
Negative	4 (3.1%)	23 (3.1%)	
Positive (≥10%)	3 (2.3%)	30 (4.0%)	
Unknown	123 (94.6%)	690 (92.9%)	
Stage			<0.001
IA	25 (19.2%)	314 (42.3%)	
IB	17 (13.1%)	91 (12.2%)	
IIA	49 (37.7%)	157 (21.1%)	
IIB	17 (13.1%)	87 (11.7%)	
IIIA	13 (10%)	66 (8.9%)	
IIIB	9 (6.9%)	28 (3.8%)	

Data are reported as number (%), mean, and median. ER: Estrogen receptor status. Student's t-test, chi-squared test, and Mann-Whitney test were used for statistical analysis.

Among patients with ER-positive tumors, 385 (51.8%) received only adjuvant hormone therapy; 293 (39.4%) adjuvant hormone and chemotherapy; 46 (6.2%) hormone, chemotherapy, and trastuzumab; 15 (2.0%) received only chemotherapy; three received chemotherapy and trastuzumab; and one, only trastuzumab. A total of 557 (75.0%) patients received adjuvant radiotherapy.

Among patients with ER-negative tumors, 102 (78.5%) received only adjuvant chemotherapy and 28 (21.5%) received chemotherapy and trastuzumab. A total of 93 (71.5%) patients received adjuvant radiotherapy.

### Survival analysis

**Figure 1:**
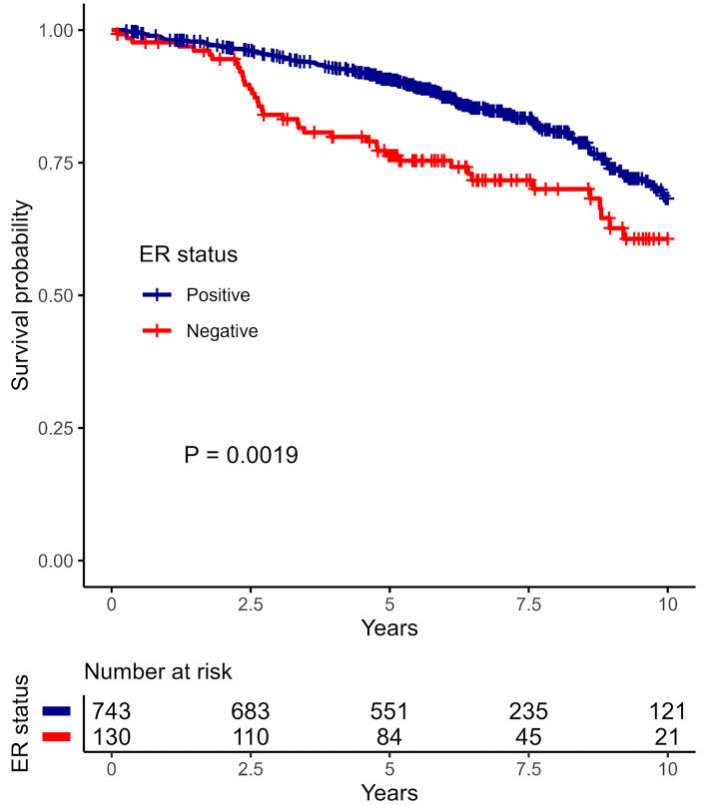
Survival curves by estrogen receptor (ER) status.

Figure 1 shows the estimated survival curves by ER status. In 10 years of follow-up, the estimated overall survival was 68.22% (63.05-73.81%) for women with ER-positive tumors and 60.63% (50.73-72.46%) for women with ER-negative tumors.

Table 2 shows the main risk factors for death in the cohort. The variables associated with increased risk of death were: age (P<0.0001), tumor size (P=0.024), and the number of positive lymph nodes (P<0.0001). Adjuvant treatment with trastuzumab (P=0.014) was associated with reduced death risk.

**Table 2:** Cox regression for all-cause mortality.

	HR	CI	P
Age	1.03	(1.02-1.05)	<0.00001
Detected by screening	0.79	(0.56-1.12)	0.187
Tumor size	1.01	(1-1.02)	0.024
Tumor grade	1.26	(0.99-1.60)	0.057
Number of positive nodes	1.08	(1.06-1.11)	<0.00001
Tumor ER-negative	1.19	(0.41-3.41)	0.747
Adjuvant hormone therapy	0.67	(0.24-1.86)	0.440
Adjuvant chemotherapy	0.91	(0.67-1.24)	0.544
Adjuvant trastuzumab	0.32	(0.13-0.69)	0.013

HR: Hazard ratio; CI: confidence interval; ER estrogen receptor. The Cox proportional hazards model was used for statistical analysis.

### Model discrimination

We assessed the model discrimination for overall survival in 5-year and 10-year follow-ups. The 5-year Royston D was 1.61 (SE: 0.18) for the full model, 1.57 (SE: 0.22) for the ER-positive model, and 1.88 (SE: 0.34) for the ER-negative model. The 10-year Royston D was 0.90 (SE: 0.15) for the full model, 0.96 (SE: 0.18) for the ER-positive model, and 1.68 (SE: 0.37) for the ER-negative model.

**Figure 2:**
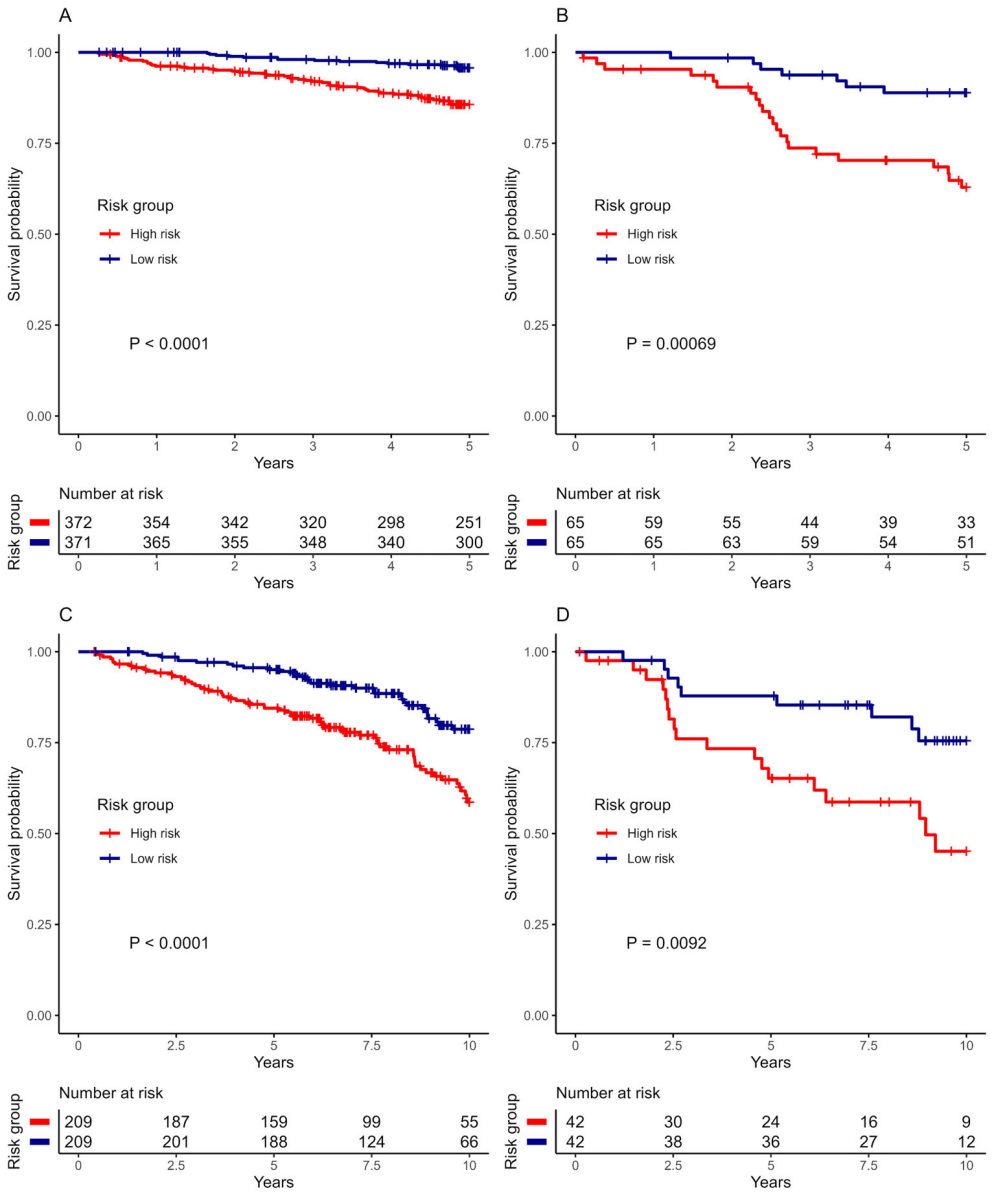
Survival curves according to the median estimated risk. **A**, Estrogen receptor (ER)-positive at 5 years. **B**, ER-negative at 5 years. **C**, ER-positive at 10 years. **D**, ER-negative at 10 years.

Figure 2 shows the survival curves for women dichotomized according to the median of estimated risk. The model discrimination was significant for ER-positive and ER-negative models at five and ten years of follow-up.

**Figure 3:**
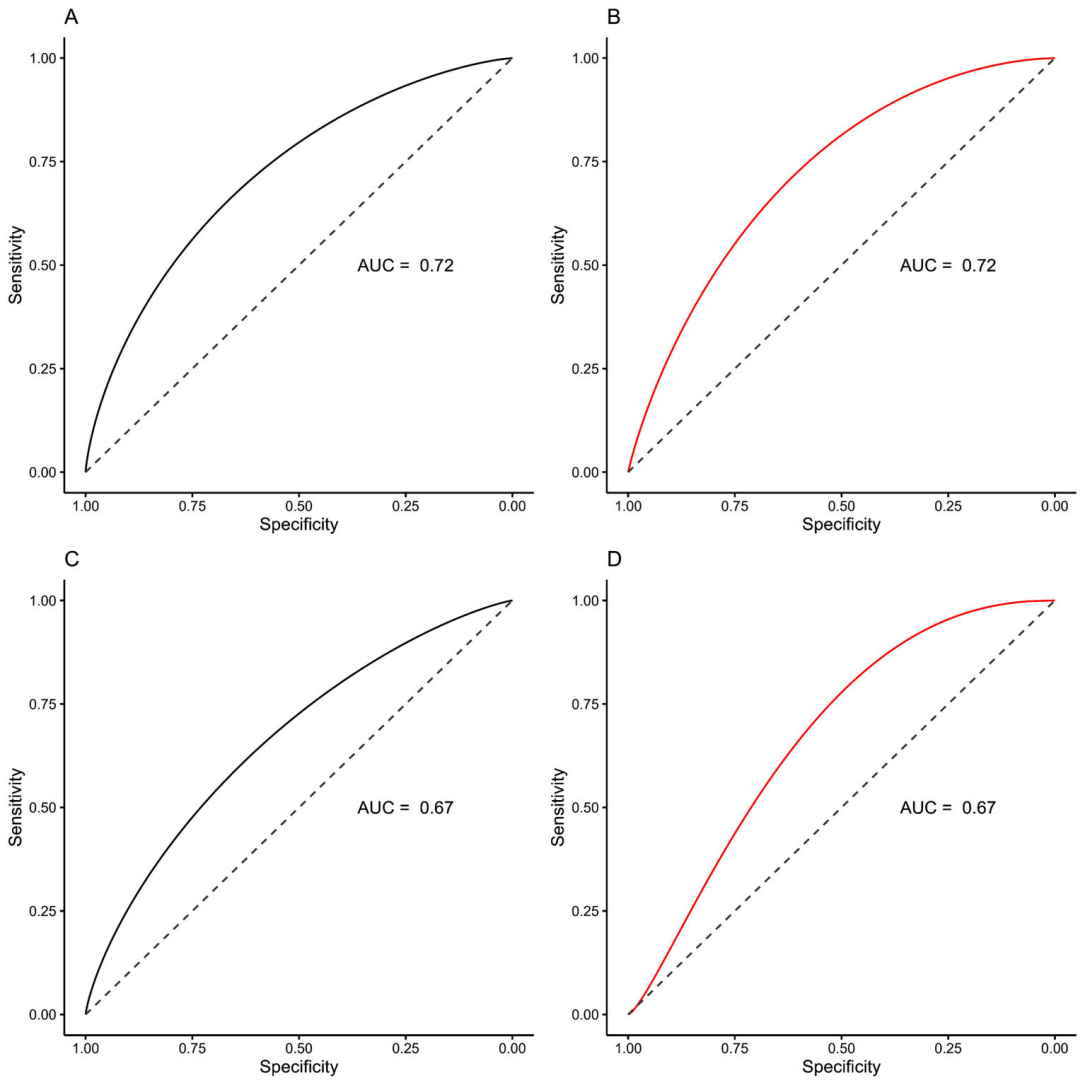
Receiver operating characteristic (ROC) curves for the model discrimination according to estrogen receptor (ER) status. **A**, ER-positive at 5 years; **B**, ER-negative at 5 years; **C**, ER-positive at 10 years; **D**, ER-negative at 10 years. AUC: area under the ROC curve.

Figure 3 shows the ROC curves for the model discrimination according to ER at five and ten years of follow-up. The area under the curve (AUC) was 0.72 (95%CI: 0.66-0.78) for the ER-positive model at 5 years, 0.72 (95%CI: 0.62-0.81) for the ER-negative model at 5 years, 0.67 (95%CI: 0.61-0.72) for the ER-positive model at 10 years, and 0.67 (95%CI: 0.54-0.77) for ER-negative model at ten years.

### Model calibration

The mean predicted survival at five years of follow-up was 90% (95%CI: 89-91%), and the observed survival at five years was 89% (95%CI: 87-91%) for the entire cohort. The mean predicted survival at ten years of follow-up was 79% (95%CI: 77-80%), and the observed survival was 76% (95%CI: 72-80%).

Supplementary Table S1 shows the predicted and observed survival at five and ten years of follow-up according to ER status, age group, tumor size, positive lymph nodes, and adjuvant treatment. The model had excellent calibration for women with ER-positive tumors treated with hormone therapy. The model overestimated the survival for women with ER-negative tumors and women treated only with chemotherapy.

**Figure 4:**
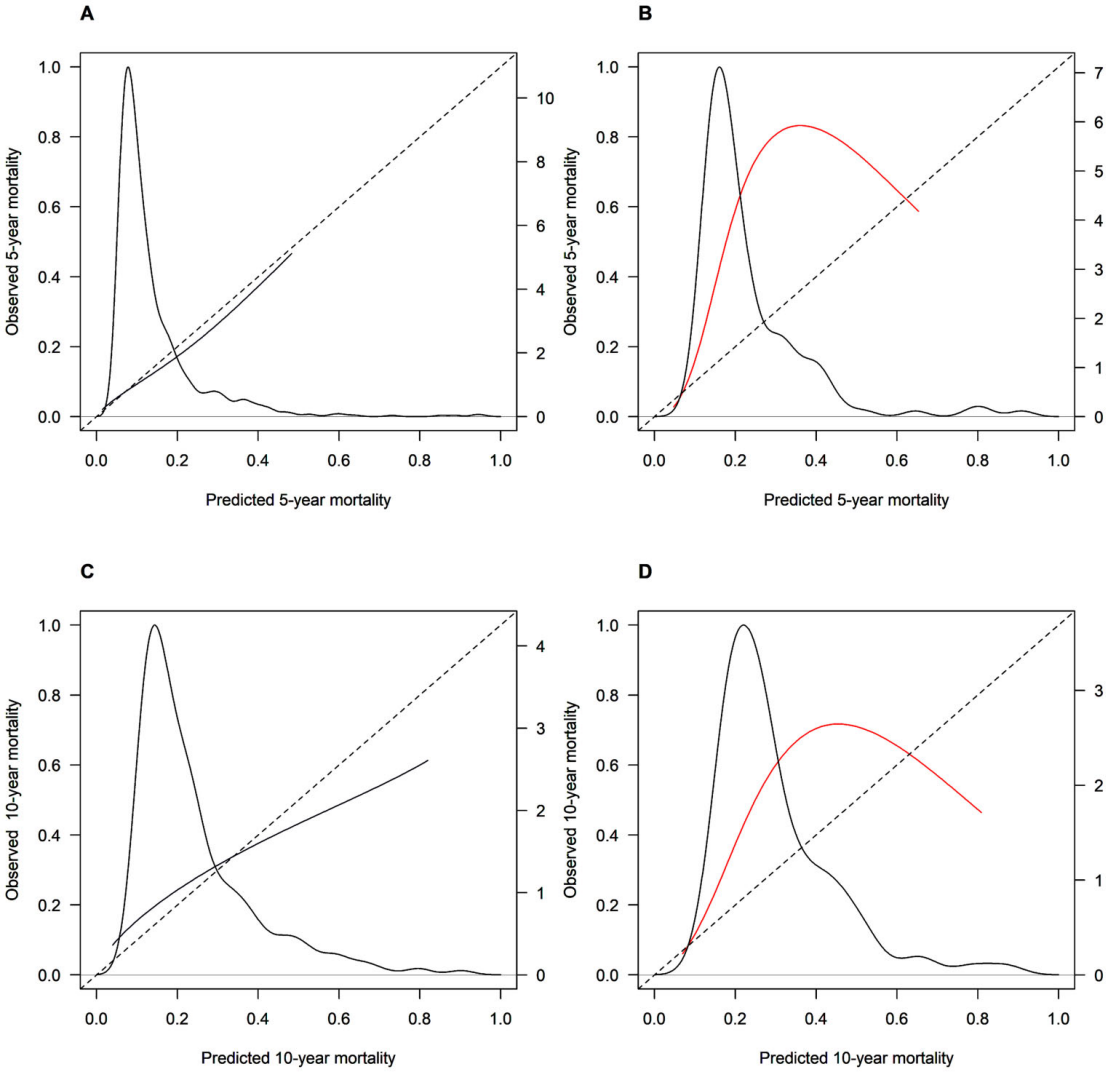
Model calibration according to estrogen receptor (ER) status. **A**, ER-positive at 5 years; **B**, ER-negative at 5 years; **C**, ER-positive at 10 years; **D**, ER-negative at 10 years.

Figure 4 shows the model calibration according to ER status and the predicted mortality at five and ten years of follow-up. The ICI was 0.005 for ER-positive 5-year mortality, 0.084 for ER-negative 5-year mortality, 0.051 for ER-positive 10-year mortality, and 0.094 for ER-negative 10-year mortality.

## Discussion

We assessed the discrimination and calibration of the PREDICT model in a well-documented cohort of early breast cancer. The model presented excellent discrimination for survival at five and ten years. The calibration was excellent for patients with ER-positive tumors treated with adjuvant hormone therapy. The model overestimated the benefits of chemotherapy and survival for patients with ER-negative tumors.

The baseline characteristics of our cohort were similar to those of other studies that previously validated the PREDICT model (7,12). The cohort used to build the PREDICT tool had 83% ER-positive tumors (5), and our cohort had 85% ER-positive tumors. Fifteen percent of ER-positive and 33% of ER-negative tumors were HER2-positive in our cohort. There were between 4 and 24% of HER2-positive tumors in ER-positive tumors and between 16 and 34% in ER-negative tumors in the cohorts used to validate the addition of HER2 to the model (13).

The main prognostic factors of our study for survival were similar to published data. Several studies in the last three decades have associated the number of positive axillary lymph nodes, tumor size, tumor grade, age at diagnosis, and tumor ER expression with survival in breast cancer patients (14).

The validation of a prognostic model is essential to establish its use in a specific population (15). The adequate sample size of a cohort depends on the number of events observed during the follow-up. We had a total of 192 deaths documented, 39 in women with ER-negative tumors and 153 in women with ER-positive tumors. The external validation of prediction models requires a minimum of 100 and, ideally, 200 or more events (16).

We demonstrated that the PREDICT model discriminates well in women who survive in five and ten years of follow-up by three methods: Royston D, survival curves, and AUC from ROC curves. The results are similar to other external validation studies of the PREDICT model (17-21). According to general guidelines, models with AUC between 0.7 and 0.8 have acceptable discrimination for clinical use (22).

The calibration of the model was better for patients with ER-positive tumors. For ER-positive tumors, the model predicted 91% survival in five years and we observed 90.7%. As observed in Figure 4A, the concordance was well distributed across the spectrum of risks. In ten years of follow-up, the model predicted 79.3% survival and we observed 77.2%. As shown in Figure 4C, there was an underestimation of mortality rate in high-risk ER-positive tumors, but there was a small proportion of cases in this group. In women with ER-negative tumors, the model underestimated the mortality rate at five and ten years. The main reason appears to be the overestimation of the effect of chemotherapy. Among the women who received only chemotherapy as adjuvant treatment, the predicted survival exceeded the observed survival in 14% at five years and 11.6 % at 10 years. In our cohort, 47% of patients received chemotherapy, which is higher than the 33% in the original study (5). The data suggested that the effect of chemotherapy on overall survival in our series was lower than that observed in clinical trials. One limitation of our study was the low number of tumors with the quantification of Ki67. The inclusion of Ki67 in the PREDICT tool improved the original model's discrimination and calibration (19). The absence of Ki67 might also be associated with the observed lack of calibration.

In conclusion, PREDICT was accurate in estimating the overall survival and the benefit of hormone therapy of women with ER-positive breast tumors in a Brazilian population. The model overestimated the overall survival and the benefit of chemotherapy of women with ER-negative tumors. New studies with more patients with ER-negative tumors and with known Ki67 are needed to conclude about the properties of the tool in this subgroup.

## Supplementary Material

Click here view [pdf].
